# Reactive Oxygen Species–Induced Modifications of Fibrin Clots as a Link Between Immune Responses and Atherothrombosis in Systemic Lupus Erythematosus

**DOI:** 10.1002/art.43371

**Published:** 2025-12-28

**Authors:** Matteo Becatti, Giacomo Emmi, Alessandra Bettiol, Amanda Mannucci, Flavia Rita Argento, Eleonora Fini, Serena Borghi, Francesca Nencini, Maria Nicastro, Irene Mattioli, Elena Silvestri, Augusto Vaglio, Domenico Prisco, Claudia Fiorillo

**Affiliations:** ^1^ Department of Experimental and Clinical Biomedical Sciences “Mario Serio,” University of Firenze Firenze Italy; ^2^ Department of Medical, Surgical and Health Sciences, University of Trieste and Clinical Medicine and Rheumatology Unit, Cattinara University Hospital, Trieste, Italy, and Centre for Inflammatory Diseases, Monash University Department of Medicine Monash Medical Centre Melbourne Clayton Victoria Australia; ^3^ Department of Medicine and Surgery, University of Parma and Unit of Occupational Medicine and Industrial Toxicology University Hospital Parma Medical Center Parma Italy; ^4^ Department of Experimental and Clinical Medicine Careggi University Hospital Florence Italy; ^5^ Department of Experimental and Clinical Biomedical Sciences “Mario Serio,” University of Firenze and Nephrology and Dialysis Unit Meyer Children's Hospital IRCCS Firenze Italy

## Abstract

**Objective:**

Cardiovascular events are major determinants of morbidity and mortality in systemic lupus erythematosus (SLE), particularly in patients with renal involvement. Although oxidative stress has been implicated in driving vascular and renal damage in SLE, the specific mechanisms remain unclear. This study investigated the potential role of oxidative stress‐induced alterations in fibrinogen structure and function in the pathogenesis of atherothrombosis in SLE.

**Methods:**

In this cross‐sectional study, we enrolled 144 adult patients with SLE and 90 matched controls. We measured blood leukocyte reactive oxygen species (ROS) production, systemic redox status, and the structural and functional features of purified fibrinogen. Correlations between these parameters and disease activity were also investigated. In vitro experiments to clarify the causal relationships among ROS levels, protein oxidation, and fibrin abnormalities provided mechanistic insights of the observed alterations.

**Results:**

Patients with SLE showed increased leukocyte ROS production, mainly due to neutrophil NADPH oxidase activation. Interestingly, renal biopsies from patients with SLE with active proliferative lupus nephritis exhibited overexpression of the NADPH oxidase enzyme complex p22phox. This was accompanied by plasma oxidative stress as indicated by elevated plasma lipid peroxidation and reduced antioxidant defenses. Fibrinogen oxidation was associated with structural and functional changes, leading to the formation of denser fibrin networks with lower clot porosity and reduced susceptibility to plasmin‐mediated fibrin lysis. Interestingly, these fibrinogen modifications correlated with alterations in redox status and disease activity.

**Conclusion:**

Oxidative stress may drive structural and functional modifications of fibrinogen in SLE, potentially acting as a novel pathogenetic mechanism in atherothrombosis among these patients.

## INTRODUCTION

Systemic lupus erythematosus (SLE) is a prototypical autoimmune disease that affects multiple organ systems and often leads to severe complications such as renal failure, neurologic disturbances, cytopenia and a range of systemic symptoms. Compared with the general population, patients with SLE carry a 2‐ to 10‐fold higher risk of cardiovascular events encompassing both arterial and venous complications.^1^, ^2^ This heightened risk is attributable not only to traditional cardiovascular risk factors but also to renal disease, which is prevalent in this patient population. Renal impairment in SLE contributes to an increased burden of cardiovascular morbidity, as it is associated with hypertension, dyslipidemia, and systemic inflammation, all of which are critical contributors to cardiovascular pathology. In addition to renal disease, genetic predisposition,^3^ soluble factors (eg, autoantibodies, proinflammatory cytokines) and autoreactive leukocytes^4^ contribute to endothelial damage in SLE.

Among nontraditional cardiovascular risk factors, oxidative stress, secondary to increased reactive oxygen species (ROS) production and/or decreased antioxidant defenses, has been suggested as possibly implicated in the pathogenesis of cardiovascular events in SLE^1^, ^2^ and positively correlated with SLE clinical activity scores.^5^ Accordingly, in patients with SLE, increased amounts of the lipid peroxidation marker malondialdehyde (MDA) were found in plasma^6^ and erythrocytes.^7^

Studies from our group recently demonstrated that leukocyte‐derived ROS can promote oxidative‐mediated alterations in the structure and function of fibrinogen, a key protein bridging inflammation and coagulation, thereby increasing prothrombotic risk in various inflammatory and noninflammatory conditions.^8^, ^9^, ^10^, ^11^, ^12^, ^13^

In this study, we investigated the primary cellular sources of ROS in a large cohort of patients with SLE and explored the relationship between oxidative stress markers, fibrinogen structural and functional properties, and clinical features. These findings provide a deeper understanding of the pathogenetic mechanisms driving cardiovascular events in this complex disease.

## PATIENTS AND METHODS

### Population, setting and clinical assessment

The study included 144 adult patients with SLE and 90 controls, comparable in terms of age, sex, and main traditional cardiovascular risk factors (ie, smoking, body mass index [BMI], hypertension, dyslipidemia, diabetes). Patients were recruited between September 2019 and January 2023 at the Lupus Clinic of the Careggi University Hospital, Firenze, Italy. All patients were classified as having SLE according to the 2019 EULAR/American College of Rheumatology Classification Criteria for Systemic Lupus Erythematosus.^14^

Patients with other systemic autoimmune diseases, active infectious, or neoplastic conditions were excluded. Controls were recruited among blood donors. The study protocol was approved by the local Ethics committee (Comitato Etico Regionale per la Sperimentazione Clinica della Regione Toscana, Sezione Area Vasta Centro; approved on November 13, 2018; ref. no. 12804_bio) and written informed consent was obtained from all participants.

At the time of blood sample collection, all patients underwent a review of their medical history, a comprehensive laboratory work‐up, and a standardized clinical assessment of potential organ involvement, according to EULAR recommendations.^15^ SLE clinical activity at the time of blood sample collection was evaluated using the Systemic Lupus Erythematosus Disease Activity Index (SLEDAI) score, and patients were stratified in three groups: those with a SLEDAI of 0, 1 to 5, and >5.^14^ History of cardiovascular events was assessed based on retrospective medical chart review; cardiovascular events were defined based on the same definitions reported in medical records. Also, information on ongoing therapies was retrieved, with reference to the use of moderate/high‐dose glucocorticoid treatment (defined as a dose of prednisone or equivalent greater than 7.5 mg/day),^16^ immunosuppressants, and hydroxychloroquine. Patients and/or the public were not involved in the design, conduct, reporting, or dissemination plans of this research.

### Assessment of systemic redox status and NADPH oxidase activity in peripheral leukocytes

Leukocyte ROS production was measured using H_2_DCF‐DA (2.5 μM) as previously described.^8^ NADPH oxidase activity in leukocyte subsets was assessed by lucigenin‐enhanced chemiluminescence and expressed as relative luminescence units/second (RLU/s).^8^ In plasma, lipid peroxidation and total antioxidant capacity (TAC) were evaluated by thiobarbituric acid reactive substances (as MDA, nanomoles per milliliter) and oxygen radical absorbance capacity assay (as Trolox equivalents, micromolar), respectively.^8^

### Fibrinogen purification, fibrinogen oxidation, structure, and function analysis

Blood samples collected in trisodium citrate or EDTA were used for fibrinogen purification as previously described^8^ and determination of its concentration and purity. We observed no differences in fibrinogen concentration and purity between patients and controls.

To evaluate fibrinogen oxidation, dityrosine fluorescence (Excitation: 316 nm; Emission: 367 nm) was assayed and normalized to protein concentration. To assess protein secondary structure, we recorded circular dichroism (CD) spectra of purified fibrinogen (0.5 mg/mL) as previously described.^8^ To provide information on three‐dimensional (3D) conformational changes of purified fibrinogen, intrinsic fluorescent spectra of the protein were acquired.^8^ We finally analyzed fibrin clots by confocal microscopy and by stimulated emission depletion (STED) superresolution, as previously reported.^17^

### Fibrinogen functional analysis: thrombin‐catalyzed fibrin polymerization and fibrin susceptibility to plasmin‐induced lysis

The functional analysis of fibrinogen included assessing the kinetics of thrombin‐induced fibrin polymerization and evaluating fibrin's susceptibility to plasmin‐mediated lysis. Thrombin‐induced fibrin polymerization was initiated and monitored according to previously established methods.^8^ Fibrin digestion with plasmin was performed as previously described,^8^ and data were expressed as the *ratio* between the densitometric reading of the purified protein at a given digestion time and that of the undigested protein (time 0 of incubation with plasmin).

### SLE renal biopsy analysis for NADPH p22phox expression

Kidney biopsies from 10 patients with lupus nephritis were analyzed.^18^ Tissue expression of p22phox, a key NADPH oxidase subunit, was evaluated by immunohistochemistry and confocal immunofluorescence. Cell‐specific colocalization was assessed using Manders’ coefficients.^19^ Full experimental details are provided in the Supplementary Materials and Supplementary Table S1.

### Investigating oxidative stress‐induced thrombus formation: in vitro studies

In another set of experiments, increasing concentrations (0.5–2 mM) of 2,2'–azobis(2–amidinopropane) dihydrochloride (AAPH) were incubated at 37°C for 12 hours with 1 mg of purified human fibrinogen (Sigma) dissolved in 1 mL phosphate buffered saline pH 7.4. To evaluate the potential preventive effect of Trolox on the afore‐mentioned AAPH‐induced oxidation reaction, 1 mg of purified human fibrinogen (Sigma) dissolved in 1 mL phosphate buffered saline pH 7.4 was incubated with 1 mM AAPH in the presence of 0.1 mM Trolox at 37°C for 12 hours. The obtained samples were used for functional and structural analyses.

### Statistical analysis

Continuous variables are presented as mean ± SD or as median and interquartile range (IQR), according to data distribution. Categorical variables are presented as n (%). All experiments were performed in triplicate, and, for each case, the mean of the three experiments ± SD was considered, after having confirmed the low intraexperiment and interexperiment variability and the reproducibility of measures using the analysis of variance (ANOVA) Bonferroni test.

Differences in continuous variables between SLE case and control groups, as well as between patients with SLE with versus those without a history of cardiovascular events, were assessed using the Student *t* or the Mann‐Whitney test for two‐group comparisons, whereas differences between subgroups of patients with SLE distinguished according to disease activity were assessed using the ANOVA or the Kruskal‐Wallis test for comparisons of more than two groups, considering if data distribution respect the normality assumption. Differences in categorical variables were assessed using the Fisher's exact test. Correlations were analyzed using Spearman's test, and ρ correlation coefficient was calculated. For the correlation between continuous and ordinal parameters, Kendall's coefficient of rank correlation was performed, and Kendall's τb was calculated. For all analyses, *P* values <0.05 were considered statistically significant. Statistical analyses were performed using the Graph Pad Prism 5 Software and the software STATA version 14. Data are available upon reasonable request by the corresponding author.

## RESULTS

### Patient populations and baseline characteristics

The demographic and clinical characteristics of the 144 patients with SLE and 90 controls are reported in Table 1. Most patients were female (126; 87.5%), with a median age at diagnosis of 35.2 years (IQR 26.4–42.8) and a median age at blood sample collection of 42.8 years (IQR 37.0–51.1). The most frequent SLE manifestations included articular (84.7%), cutaneous (57.6%), hematologic (52.8%), and renal involvement (32.6%). Approximately one‐third of patients had confirmed antiphospholipid antibodies (aPL) positivity. Of the 47 patients with renal involvement, 40 had a biopsy‐proven lupus nephritis and 15 had an associated aPL positivity. The majority of the available kidney biopsies (67%) were categorized as class III to IV (with coexisting class V in 15%) according to the International Society of Nephrology/Renal Pathology Society classification. Regarding traditional cardiovascular risk factors, the median BMI was 24 (IQR 16–42); 41% patients had hypertension, and 28.5% were current or past smokers. Dyslipidemia and diabetes were present in 16% and 6.3% of patients, respectively.

**Table 1:** Main demographic and clinical features of the patients in the SLE and control groups included in the study*

	Patients with SLE (n = 144)	Controls (n = 90)	Patients with SLE stratified according to disease activity			*P*‐value^a^
			SLEDAI 0 (n = 38)	SLEDAI 1–5 (n = 77)	SLEDAI 6+ (n = 29)	
Demographic data						
Female sex, n (%)	126 (87.5)	79 (87.8)	33 (86.8)	69 (89.6)	24 (82.8)	0.596
Age at diagnosis, median (IQR)	35.2 (26.4–42.8)		32.9 (26.3–42.1)	35.3 (26.0–45.8)	36.9 (28.9–42.9)	0.669
Age at blood sample collection, median (IQR)	42.8 (37.0–51.1)	42.0 (36–52)	44.7 (35.7–54.7)	40.4 (37.0–50.0)	44.2 (38.9–54.5)	0.380
Clinical involvement						
Disease duration, median (IQR), mo	68.9 (9.1–163.0)		100.7 (1.9–190.3)	42.5 (6.0 –155.4)	113.5 (29.0–214.9)	0.194
History of cardiovascular events, n (%)	33 (22.9)		2 (5.3)	16 (20.8)	15 (51.7)	**<0.001** ^**b**^
Articular, n (%)	122 (84.7)		30 (78.9)	68 (88.3)	24 (82.8)	0.400
Cutaneous, n (%)	83 (57.6)		22 (57.9)	42 (54.5)	19 (65.5)	0.594
Hematologic, n (%)	76 (52.8)		19 (50.0)	40 (51.9)	17 (58.6)	0.765
Renal, n (%)	47 (32.6)		11 (29.0)	25 (32.5)	11 (37.9)	0.746
Neuropsychiatric, n (%)	25 (17.4)		6 (15.8)	13 (16.9)	6 (20.7)	0.860
Cardiac, n (%)	9 (6.3)		2 (5.3)	5 (6.5)	2 (6.9)	0.955
Gastrointestinal, n (%)	2 (1.4)		0	1 (1.3)	1 (3.4)	
SLEDAI, median (IQR)	2 (0–4)					
Cardiovascular risk factors						
BMI, median (IQR)	24 (16–42)	24 (16–42)	23 (16–42)	24 (15–42)	24 (16–41)	>0.999
Hypertension, n (%)	59 (41.0)	38 (42.2)	13 (34.2)	35 (45.5)	11 (37.9)	0.480
Smoke, n (%)	41 (28.5)	26 (28.9)	11 (29.0)	22 (28.5)	8 (27.5)	0.992
Dyslipidemia, n (%)	23 (16.0)	14 (15.6)	6 (15.8)	13 (16.9)	4 (13.8)	0.927
Diabetes, n (%)	9 (6.3)	6 (6.7)	2 (5.3)	4 (5.2)	3 (10.3)	0.595
aPL positivity, n (%)	45 (31.3)		8 (21.1)	22 (28.6)	15 (51.7)	**0.026** ^**a**^
Treatments, n (%)						
Prednisone alone (dosage of >7.5mg/d)	45 (31.3)		12 (31.6)	25 (32.5)	8 (27.6)	0.059
Immunosuppressants alone or + prednisone (dosage ≤7.5 mg/d)	57 (39.6)		10 (26.3)	33 (42.9)	14 (48.3)	
Immunosuppressants + prednisone (dosage of >7.5 mg/d)	8 (5.6)		1 (2.6)	7 (9.1)	0 (0)	
Hydroxychloroquine (co)treatment	66 (45.8)		14 (36.8)	36 (46.8)	16 (55.2)	0.317

* aPL, antiphospholipid antibodies; BMI, body mass index; IQR, interquartile range; SLE, systemic lupus erythematosus; SLEDAI, Systemic Lupus Erythematosus Disease Activity Index.

^a^ From Kruskal‐Wallis or Fischer exact test comparing the three groups of patients with SLE with no, low, and moderate‐to‐high disease activity.

^b^ Statistically significant with *P* < 0.05. Bold values indicate statistically significant differences (*P* < 0.05)

At the time of blood sample collection, most patients were receiving immunosuppressants (alone or in association with prednisone ≤7.5mg/day) (39.6%) or prednisone in monotherapy, at a dosage >7.5mg/day (31.3%). Sixty‐six patients were also on hydroxychloroquine (45.8%). Considering SLE disease activity, at the time of blood sample collection, 38 patients (26.4%) were classified as having SLEDAI = 0, 77 (53.5%) as having SLEDAI 1 to 5, and 29 (20.1%) as having SLEDAI >5.

Thirty‐three patients (22.9%) had a history of cardiovascular events, including 14 with cases of deep venous thrombosis (DVT; associated with acute lower limb ischemia in two of them), nine cases of stroke or transient ischemic attack, seven of acute myocardial infarction (with subsequent DVT in two of them), one case of pulmonary embolism, one of intracardiac thrombosis, and one of acute lower limb ischemia. Cardiovascular events were significantly more frequent in patients with SLEDAI >5 (51.7%) as compared to those with SLEDAI 1 to 5 (20.8%) or SLEDAI 0 (5.3%) (*P* < 0.001 across the three groups). aPL positivity was found in 21 of the 33 patients with cardiovascular events (63.6%), as compared to 24 of 111 without cardiovascular events (21.6%). aPL positivity was significantly more frequent in patients with SLEDAI >5 (51.7%) as compared to patients with SLEDAI 0 or SLEDAI 1 to 5 (21.1% and 28.6%, respectively, *P* = 0.026). All other demographic and clinical features were comparable between SLE subgroups, stratified according to disease activity (Table 1).

### Intracellular ROS assessment and NADPH oxidase activity in peripheral leukocytes

**Figure 1:**
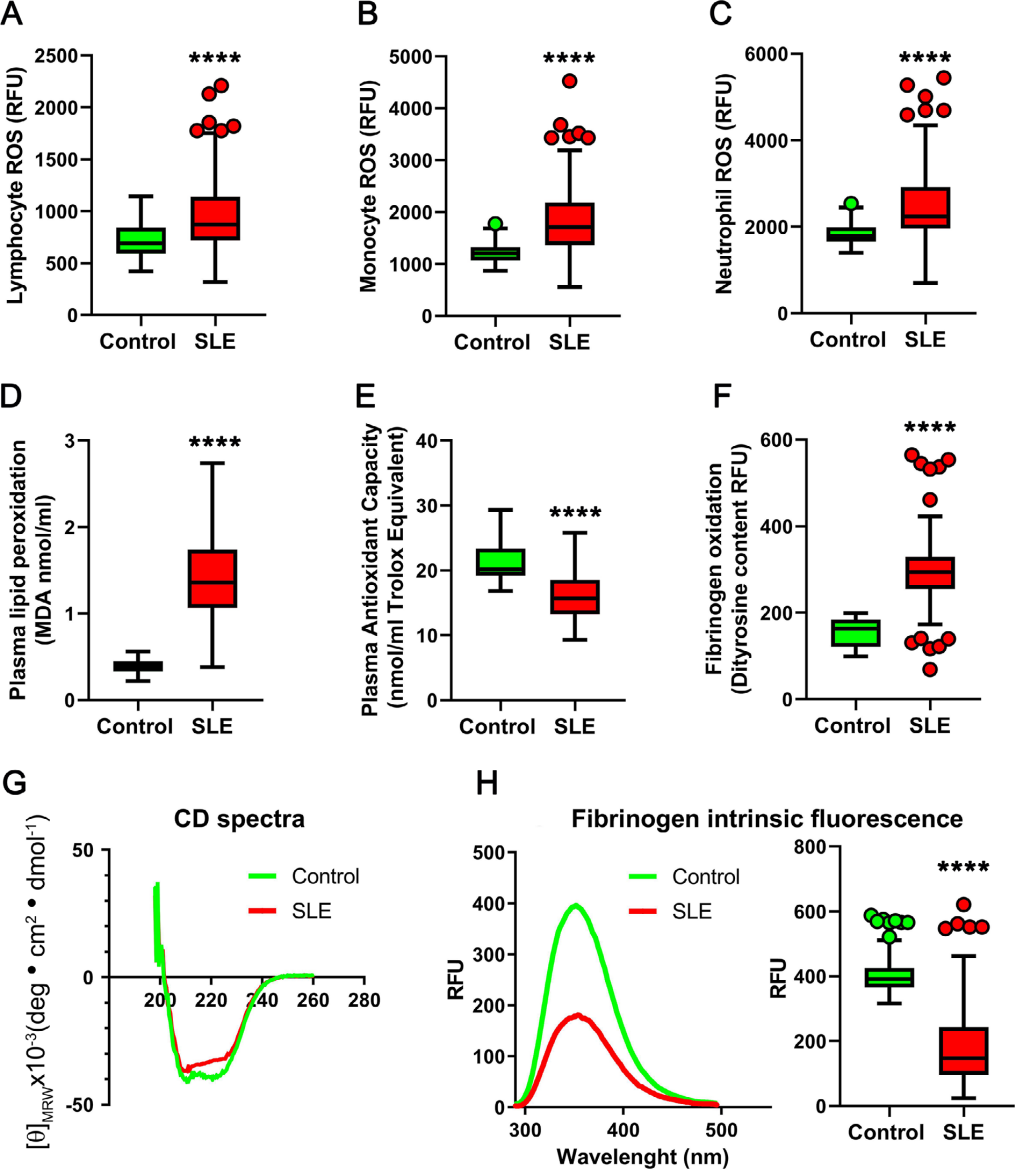
Redox status and fibrinogen structural analyses. (A–C) Lymphocyte, monocyte, and neutrophil ROS production; (D) plasma lipid peroxidation (E) and total antioxidant capacity; (F) dityrosine content; (G) representative CD spectra (H); and intrinsic protein fluorescence spectra of purified fibrinogen from patients with SLE (n = 144) and controls (n = 90). ****Statistical significance is indicated as *P* < 0.0001. CD, circular dichroism; MDA, malondialdehyde; RFU, relative fluorescence units; ROS, reactive oxygen species; SLE, systemic lupus erythematosus.

Patients with SLE showed a significant increase in ROS levels in all three leukocyte subpopulations, when compared to controls (values are median [IQR]; for lymphocyte ROS: 871 [719–1,138] vs 689.5 [594–841] relative fluorescence units [RFU] for cases and controls, respectively, *P* < 0.001 [Figure 1A]; for monocyte ROS: 1,709 [1,366–2,186] vs 1,208 [1,066–1,325] RFU, *P* < 0.001 [Figure 1B]; for neutrophil ROS: 2,237 [1961–2,915] vs 1,789 [1,655–1990] RFU, *P* < 0.001 [Figure 1C]).

NADPH oxidase activity resulted significantly increased in neutrophils from patients with SLE compared to those obtained from controls (42,432 ± 18,998 vs 13,234 ± 4,223 RLU/s; *P* = 0.001). Conversely, no significant differences in NADPH oxidase activity were observed in lymphocyte and monocyte from patients with SLE with respect to controls (Supplementary Figure S1).

### Lipid peroxidation and total antioxidant capacity estimation

In plasma, we found a significantly increased lipid peroxidation (values are median [IQR]; 1.36 [1.07–1.74] vs 0.39 [0.33–0.45] MDA nmol/mL; *P* < 0.0001) and a reduced total antioxidant capacity (15.71 [13.29–18.45] vs 20.22 [19.23–23.36] Mm Trolox equivalent; *P* < 0.0001) in patients with SLE as compared with controls (Figure 1D and E, respectively).

### Fibrinogen oxidation, structure, and function analysis

A significant increase in dityrosine content was evident in fibrinogen from patients with SLE compared to controls (values are median [IQR]; 294 [256–329] vs 163 [121–184] RFU; *P* < 0.0001) (Figure 1F). CD spectroscopy indicated, in fibrinogen purified from controls, a typical α‐helix secondary structure with minima at 208 nm and at 222 nm; conversely, a decreased negative peak in the 215‐ to 225‐nm region was observed in SLE fibrinogen, suggesting a reduction in α‐helical content (Figure 1G). Intrinsic fluorescence was also significantly reduced in SLE fibrinogen (Figure 1H), indicating altered tertiary structure.^20^

**Figure 2:**
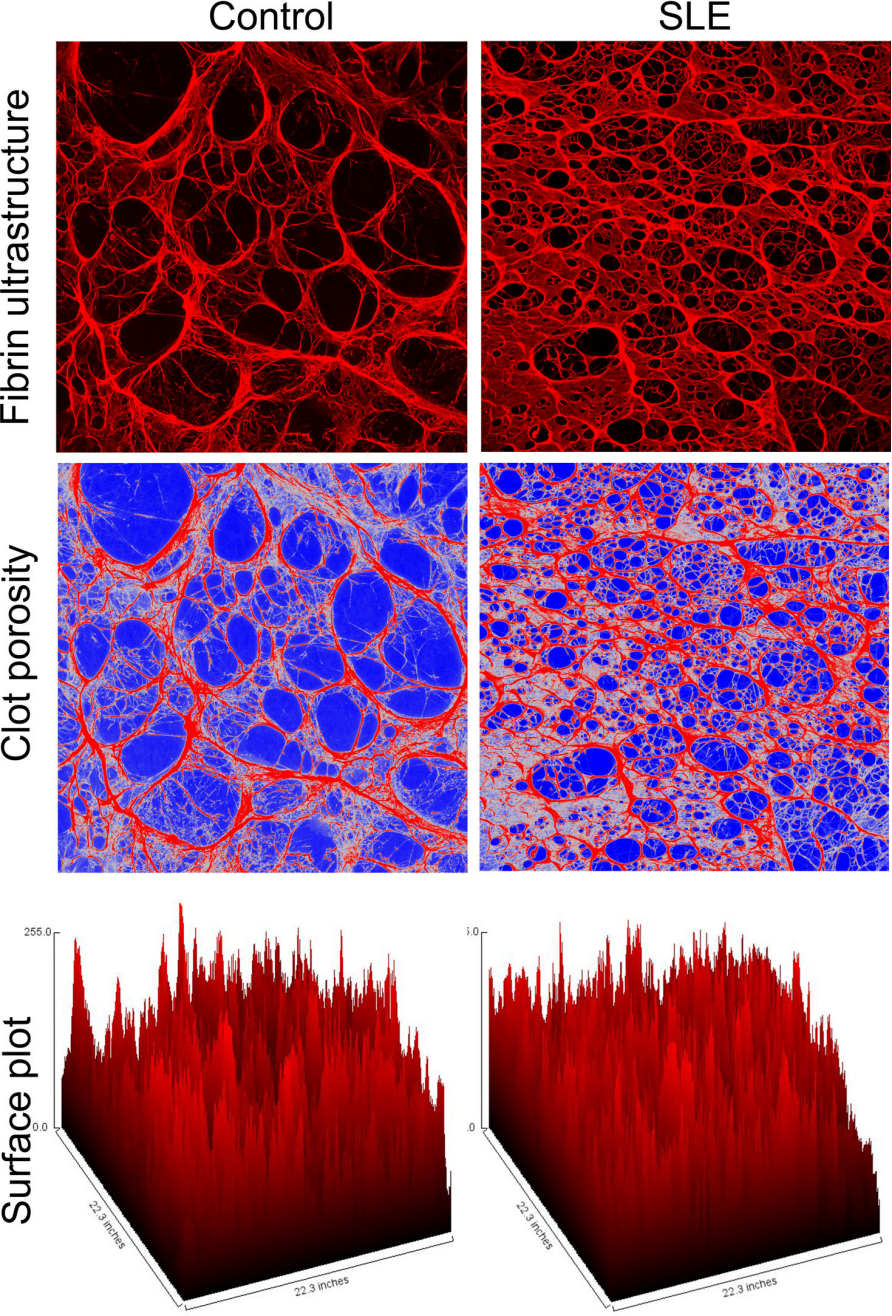
Confocal microscopy analysis of fibrin gel ultrastructure and porosity in fibrinogen samples from controls and patients with SLE. Representative high‐magnification confocal images (630×) show distinct structural differences in fibrin networks formed from purified fibrinogen of healthy controls (left column) versus patients with SLE (right column). Top panels (fibrin ultrastructure): In control samples, fibrin fibers appear thick and well‐organized and form a loose meshwork with large, well‐defined pores. In contrast, SLE‐derived fibrin gels are characterized by a denser network composed of thinner, tightly packed fibers with visibly smaller and irregular pores, indicating altered polymerization dynamics. Middle panels (clot porosity): Image processing was applied to highlight pore spaces in blue, overlaid on the red‐stained fibrin network. This visualization underscores the stark reduction in clot porosity in SLE samples, reflecting the formation of a more compact and potentially less degradable fibrin matrix. Bottom panels (surface plot): Three‐dimensional surface plots generated from fluorescence intensity data of the original confocal images demonstrate topographical differences in clot architecture. The control fibrin gel displays a highly textured surface with pronounced peaks and valleys, corresponding to thick, spaced fibers. In contrast, the SLE fibrin surface is flatter and more uniform, consistent with the presence of tightly packed, fine fibrin strands and reduced spatial variation. SLE, systemic lupus erythematosus. Color figure can be viewed in the online issue, which is available at http://onlinelibrary.wiley.com/doi/10.1002/art.43371/abstract.

Confocal microscopy analysis revealed marked structural differences between SLE and control fibrin networks, especially in fiber architecture and porosity (Figure 2). In control samples, fibrin gels displayed thick, well‐organized fibers forming a loosely arranged meshwork with large, open pores. In contrast, fibrin clots from patients with SLE exhibited a dense, compact network composed of thinner fibers and significantly smaller pores. These structural differences were further highlighted in the pseudocolored images representing clot porosity, where open spaces (in blue) were prominent in controls but drastically reduced in SLE samples. Surface plot renderings of fluorescence intensity also demonstrated a flatter, more uniform fibrin structure in SLE, consistent with tighter fiber packing and reduced topographical complexity.

**Figure 3:**
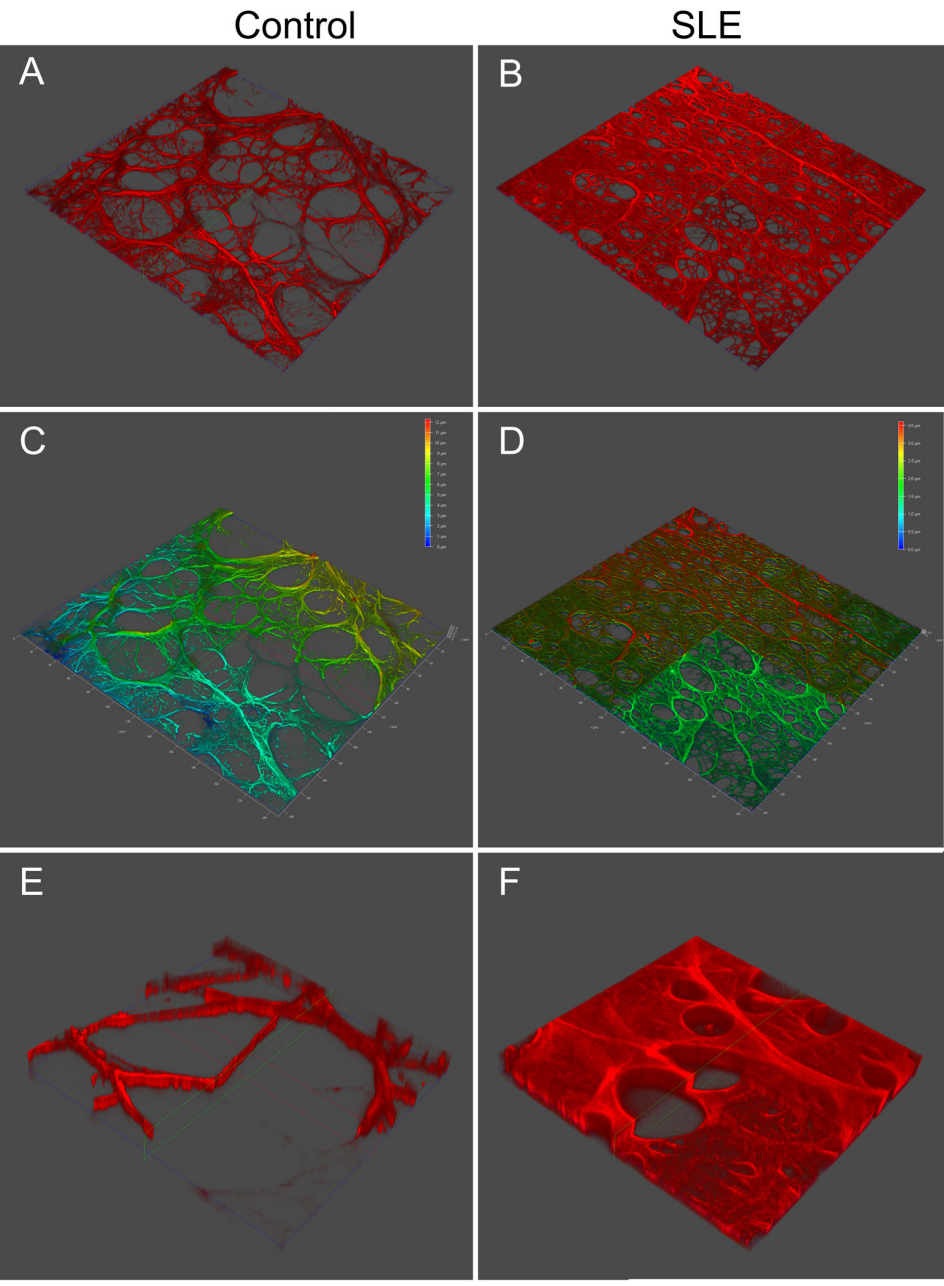
3D analysis of fibrin gels of fibrinogen purified from patients with SLE and controls. (A, B) 3D confocal microscopy analysis and (C, D) 3D clipping structure of fibrin gels of fibrinogen purified from patients with SLE and controls (630× magnification). SLE fibers were closely packed in the bulk of gel, obscuring the individual fibers and producing thin sheets with small pores. (E, F) 3D STED superresolution microscopy analysis of fibrin gels of fibrinogen purified from patients with SLE and controls (1000× magnification). STED superresolved microscopy revealed a marked increase in fiber density and clot porosity in fibrin from patient with SLE when compared to control. Leica Application Suite X Software analysis demonstrated that fibrin fibers from fibrinogen purified from patients with SLE showed a significant decrease in fiber diameter and clot porosity when compared to controls. 3D, three‐dimensional; SLE, systemic lupus erythematosus; STED, stimulated emission depletion. Color figure can be viewed in the online issue, which is available at http://onlinelibrary.wiley.com/doi/10.1002/art.43371/abstract.

**Figure 4:**
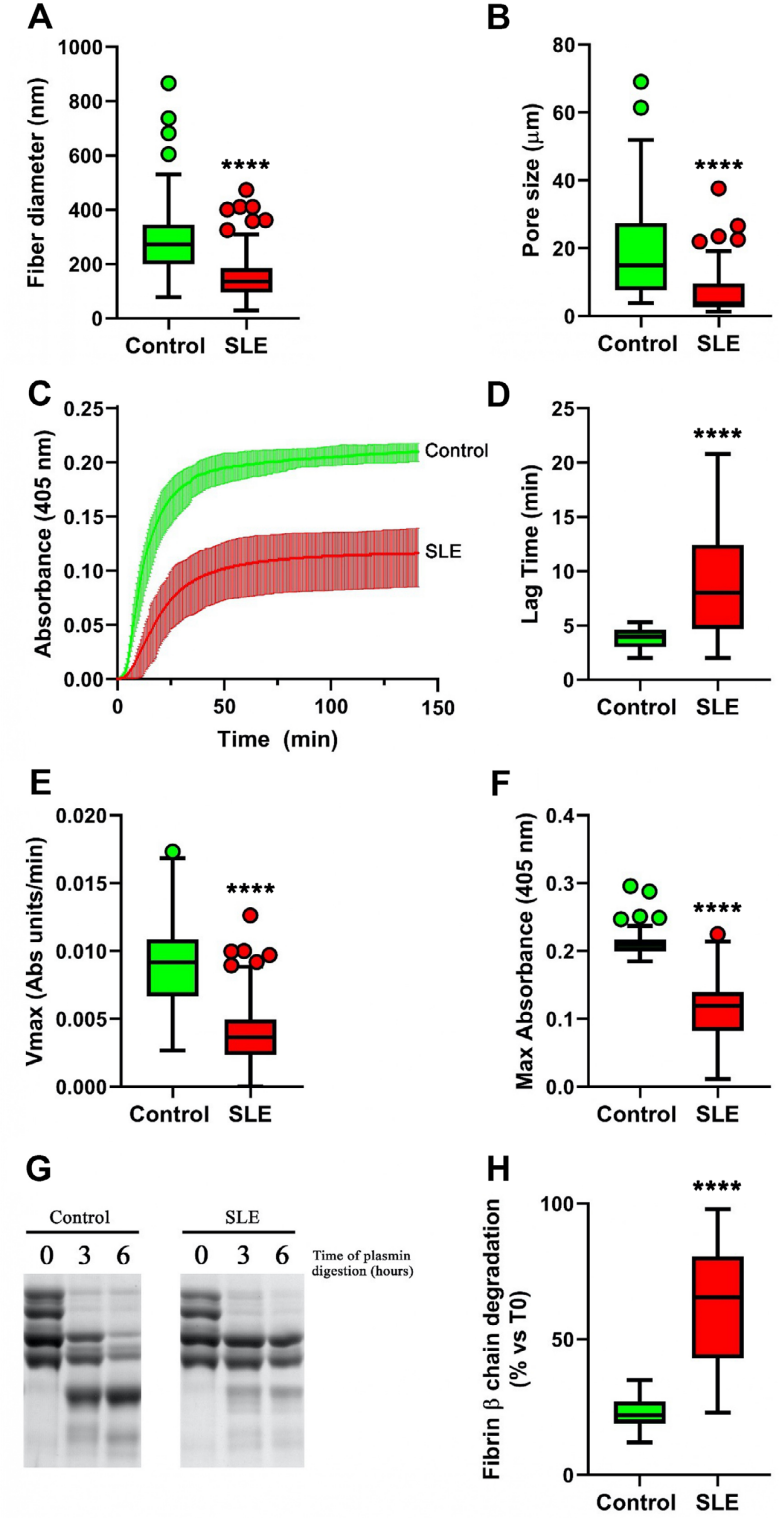
Fibrin structural and functional analyses. (A) Fibrin fiber diameter (B) and pore size of purified fibrinogen from patients with SLE (n = 144) and controls (n = 90). (C) Thrombin‐catalyzed fibrin polymerization curves and (D) corresponding lag phase, (E) Vmax, (F) and Max Abs were assessed in the same samples. (G) Representative gel of fibrin lysis after zero to six hours of plasmin incubation (H) and quantification of residual fibrin β chain after six hours of plasmin digestion in both groups. Statistical significance is indicated as ****P < 0.0001. Ab, antibody; SLE, systemic lupus erythematosus; Max, maximum; Vmax, maximum velocity. Color figure can be viewed in the online issue, which is available at http://onlinelibrary.wiley.com/doi/10.1002/art.43371/abstract.

To further assess the 3D organization of fibrin gels, we performed 3D confocal volume reconstruction and STED microscopy (Figure 3). STED microscopy is a superresolution imaging technique that surpasses the diffraction limit of conventional confocal microscopy, achieving lateral resolutions down to 20 to 30 nm. Unlike standard confocal microscopy, which is limited to a resolution of approximately 200 nm due to the diffraction of light, STED employs a secondary depletion laser that selectively deactivates fluorophores at the periphery of the excitation spot. This process effectively narrows the point of fluorescence emission, allowing for much finer spatial resolution. In our study, STED imaging enabled precise visualization and quantification of individual fibrin fibers, their diameters, and network branching patterns, which would not be resolvable at this level using confocal microscopy alone. This enhanced resolution was critical in revealing the ultrastructural abnormalities here observed, such as thinner, more tightly packed fibrin fibers in SLE samples compared to controls, which underlie the prothrombotic characteristics of oxidatively modified clots. In Figure 3, panels A and B show 3D volume renderings of control and SLE clots, illustrating the dense and closely packed fiber network in SLE. Clipping analysis with height‐based pseudocoloring (panels C and D) confirmed the tighter fibrin mesh in SLE samples, with reduced pore volume and regions displaying a gel‐like structure, suggestive of an altered polymerization process and compromised spatial heterogeneity. Whereas Figure 2 provides a high‐resolution two‐dimensional view of the fibrin ultrastructure, Figure 3 offers complementary and spatially richer 3D data that confirm and extend these findings by revealing differences in internal clot organization, depth, and nanoscale features not visible in traditional confocal projections. Finally, high‐resolution 3D STED microscopy (panels E and F) allowed direct visualization of individual fiber diameters and pore morphology. In SLE‐derived samples, fibers were notably thinner, and the network appeared uniformly compact with minimal spacing between filaments. These qualitative findings were supported by quantitative analysis, demonstrating a significant reduction in fiber diameter (Figure 4A) and clot porosity (Figure 4B) in SLE.

### Fibrinogen functional analysis: thrombin‐catalyzed fibrin polymerization and fibrin susceptibility to plasmin‐induced lysis

Fibrinogen from patients with SLE showed a reduced ability to polymerize into fibrin as compared with that from controls (Figure 4C). Likewise, we observed significant differences in the main parameters of the fibrin polymerization process (lag phase, maximum velocity [Vmax], and maximum absorbance [Max abs]) between patients with SLE and controls (Figure 4D–F). An impaired fibrin susceptibility to plasmin‐induced degradation (Figure 4G), as revealed by the quantification (values are median % [IQR]) of the residual fibrin β chain after six hours of plasmin digestion, indicated the presence of fibrin resistance to plasmin‐induced lysis in SLE (densitometric value of 66% [43–80] vs 22% [19–27] at T0 in SLE and control groups, respectively; *P* < 0.0001) (Figure 4H).

### Correlation between fibrinogen functional/structural features and redox status

**Figure 5:**
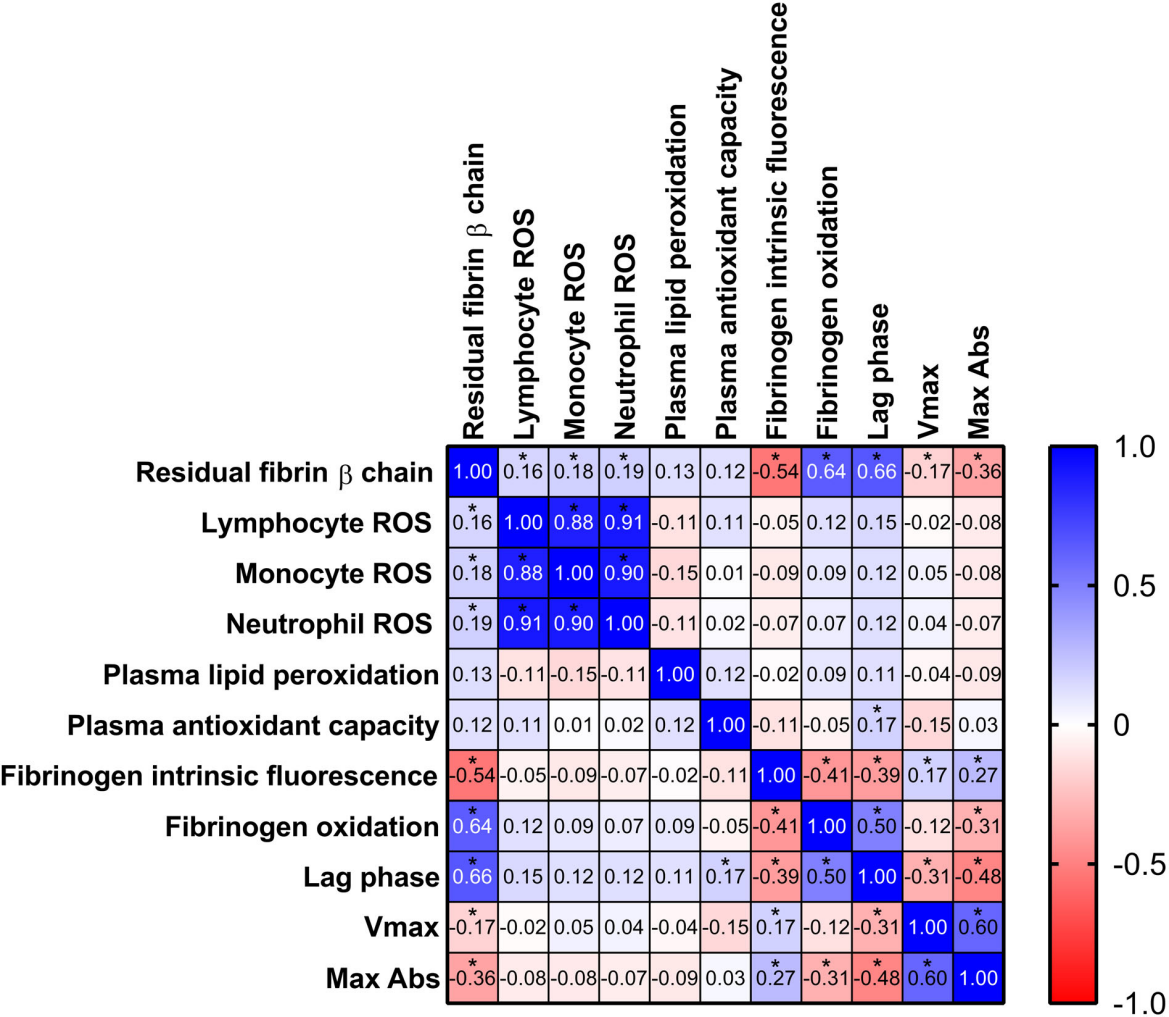
Correlation matrix of the redox status parameters in patients with systemic lupus erythematosus. *Statistically significant for *P* < 0.05. Max Abs, maximum absorbance; ROS, reactive oxygen species; Vmax, maximum velocity. Color figure can be viewed in the online issue, which is available at http://onlinelibrary.wiley.com/doi/10.1002/art.43371/abstract.

Correlation analyses among redox parameters and fibrinogen structural and functional features are reported in Figure 5. Fibrin resistance to plasmin‐induced lysis showed positive correlations with ROS levels in lymphocyte, monocyte, and neutrophil, as well as with fibrinogen oxidation marker (dityrosine content) and functional alterations in fibrin polymerization parameters (lag phase, Vmax, Max Abs). Notably, a strong association with intrinsic fibrinogen fluorescence further indicates that tertiary structural changes play a key role in enhancing clot resistance to degradation. Overall, these findings suggest that oxidative stress promotes the formation of structurally altered, lysis‐resistant fibrin clots, contributing to the prothrombotic phenotype observed in SLE. The central role of fibrinogen oxidation in driving structural and functional alterations is underscored by the strong correlations observed between dityrosine content, a marker of fibrinogen oxidation, and both intrinsic fluorescence, which reflects changes in the protein's tertiary structure, and key parameters of fibrin polymerization dynamics (lag phase and Max Abs). These associations highlight that oxidative modifications not only disrupt fibrinogen's conformation but also impair its ability to properly polymerize into fibrin, thereby contributing to the formation of aberrant, prothrombotic clots in SLE (Figure 5).

When stratifying patients with SLE according to disease activity, higher SLEDAI scores were associated with increased levels of intracellular ROS. Conversely, we observed no difference among the three SLE groups in terms of fibrinogen degradation, fibrin polymerization, plasma lipid peroxidation, and plasma total antioxidant capacity (Supplementary Table S2).

These results were confirmed also when assessing the correlation between SLEDAI (as ordinal score) and the considered redox parameters (Supplementary Table S2). To explore the possible impact of treatment on this relationship, we performed stratified analyses according to the treatment category (none or prednisone alone; immunosuppressants with or without prednisone). No statistically significant correlation emerged when assessing the association between SLEDAI and redox parameters according to treatment subgroups, likely due to a low statistical power on smaller sample sizes (data not shown). Also, no statistically significant differences emerged in the levels of ROS and fibrinogen‐related parameters in patients with versus without history of cardiovascular events (Supplementary Table S3).

### p22phox expression in renal biopsies

p22phox is an essential membrane subunit that plays a central role in NADPH oxidase complex assembly and activation. An intense expression of p22phox was found in all the 10 examined renal biopsies from patients with SLE with active proliferative forms of lupus nephritis, (ie, classified as class III–IV nephritis according to the 2018 International Society of Nephrology/Renal Pathology Society revised classification).^21^ Immunohistochemical analysis showed that the tissue expression of p22phox was detectable mainly within the interstitial periglomerular and peritubular inflammatory infiltrates but also in some glomeruli, mainly those showing inflammatory cell infiltration (Supplementary Figure S2).

In agreement with the systemic redox profile observed in patients with SLE, confocal microscopy analysis of renal tissue revealed that CD15^+^ neutrophils were the predominant cellular source of p22phox expression (Supplementary Figure S3). This NADPH oxidase subunit, critically involved in ROS generation, showed strong colocalization with the neutrophil marker CD15, with a colocalization rate of 76% (Supplementary Table S4).

### Investigating oxidative stress‐induced thrombus formation: in vitro studies

Quantification of residual β‐chain intensity after six hours of plasmin digestion of human purified fibrinogen treated with AAPH (a chemical compound with pro‐oxidant properties), in the absence or presence of 0.1 mM Trolox, is reported in Supplementary Figure S4A. Fibrin clots incubated with increasing AAPH concentrations showed reduced susceptibility to plasmin‐induced lysis at each considered time of plasmin digestion (Supplementary Figure S4B).

As shown in Supplementary Figure S4A, the simultaneous incubation of AAPH and Trolox (a water‐soluble derivative of vitamin E with antioxidant properties) was able to prevent the observed changes in fibrin digestion by plasmin. ROS‐induced fibrinogen oxidation was assessed by measuring dityrosine content on purified fibrinogen fractions (Supplementary Figure S4C).

In an in vitro fibrinogen polymerization assay, in the presence of increasing AAPH concentrations, lag time increased in a dose‐dependent manner (Supplementary Figure S4E), whereas Vmax and Max absorbance progressively and significantly decreased (Supplementary Figure S4F and G). When 0.1 mmol/L Trolox was added to the AAPH incubation reactions and thrombin‐catalyzed fibrinogen polymerization was performed, the observed changes were prevented (Supplementary Figure S4D).

When studying fibrinogen structure, in AAPH‐treated fibrinogen, a decreased negative peak in the 205‐ to 225‐nm region was observed, suggesting a reduced α‐helical content, which was prevented by the simultaneous incubation of AAPH with Trolox (Supplementary Figure S4H). Fibrinogen intrinsic emission fluorescence (tertiary structure) decreased in an oxidation‐dependent manner, and Trolox treatment prevented these changes, demonstrating the key role of oxidation in fibrinogen tertiary structure modification (Supplementary Figure S4I).

Confocal microscopy analysis revealed that fibrin gels from oxidized fibrinogen were much denser, with narrow pores and thin fibers when compared to those from controls, which presented larger pores and thicker fibers (Supplementary Figure S4L). Moreover, AAPH 2‐mM–treated fibrinogen showed an unpolymerized fibrin, in accordance with the polymerization assay (Supplementary Figure S4D). Trolox treatment prevented these changes, demonstrating the key role of oxidation in fibrin gel structure modification (Supplementary Figure S4L).

## DISCUSSION

Arterial and venous thrombosis are well‐established complications of SLE, with a prevalence that can exceed 50% in high‐risk patients, representing a major cause of morbidity and mortality, even among young individuals. Beyond traditional cardiovascular risk factors, SLE‐specific elements such as disease activity and organ involvement (notably lupus nephritis) have been implicated in the development of atherothrombosis.^22^, ^23^, ^24^

Although a few studies have reported systemic redox imbalance in active SLE,^25^, ^26^, ^27^ the precise mechanisms linking oxidative stress to vascular and renal damage remain unclear.^28^ Increasing evidence supports the role of sustained ROS production in the pathogenesis of autoimmune diseases.^8^, ^29^, ^30^, ^31^ Interestingly, some genetic variants associated with reduced NOX2‐derived ROS levels appear to predispose to SLE rather than protect against it.^32^

Here, we provide new insights into the complex role of oxidative stress in SLE, focusing on how ROS‐mediated structural and functional alterations of fibrinogen may contribute to thrombotic risk. We confirm a marked redox imbalance in patients with SLE,^33^, ^34^ particularly an upregulation of NADPH oxidase activity in circulating neutrophils, a major physiologic sources of ROS. Neutrophils in SLE are known to be hyperactivated, exhibit altered phagocytic function, and spontaneously release neutrophil extracellular traps (NETs), all of which promote a prothrombotic environment.^35^

Oxidation is known to be responsible for protein conformational changes and might lead to (auto)‐antigen generation, which in turn drive inflammation, tissue damage, immune profile changes, and autoimmune response.^36^, ^37^ Our findings demonstrate that increased oxidative stress is tightly associated with fibrinogen oxidation, resulting in significant structural and functional alterations. These include altered secondary and tertiary structure of SLE fibrinogen, as shown by far‐UV CD and intrinsic fluorescence, respectively.

Using STED superresolution microscopy, an advanced technique that enables nanoscale visualization of the fibrin network, we provide the first ultrastructural visualization of SLE fibrin network, revealing thinner fibers, reduced porosity, and a more compact clot architecture, features that are known to confer resistance to plasmin‐induced lysis and are frequently observed in other autoimmune and inflammatory conditions.^13^, ^38^, ^39^ This can raise the possibility that fibrinogen oxidative modification plays a key role in this process.

We observed that fibrinogen dityrosine content, a marker of fibrinogen oxidation, inversely correlates with thrombin‐catalyzed fibrin polymerization rate, suggesting a direct effect of oxidation on fibrin polymerization.^40^, ^41^, ^42^, ^43^ The presence of oxidation‐prone residues such as proline and arginine within the thrombin‐cleavage site of fibrinogen may partly explain these alterations observed in patients with SLE.^43^ In addition, our results demonstrate that SLE fibrin clots exhibit resistance to plasmin‐induced lysis, a feature closely linked to ROS production, fibrinogen oxidation, and structural alterations. Notably, our results indicated that, in patients with SLE, parameters related to fibrinogen structural and functional features were comparable in patients with and without history of cardiovascular events, suggesting the association of SLE per se to an increased thrombotic tendency.

To validate the causal role of oxidative stress in these phenomena, we conducted in vitro experiments exposing purified fibrinogen to the ROS‐generating agent AAPH, with or without the antioxidant Trolox. ROS exposure reproduced the structural and functional abnormalities observed in SLE‐derived fibrinogen, including impaired polymerization and resistance to plasmin‐induced lysis. Co‐incubation with the antioxidant Trolox prevented these changes, confirming that ROS are directly responsible for the observed fibrinogen alterations. However, despite this mechanistic clarity, antioxidant therapies have shown some promise in alleviating oxidative stress associated with SLE, but their effectiveness in fully halting or reversing disease progression remains uncertain and is still a matter of ongoing research and debate.^36^, ^44^, ^45^, ^46^

Importantly, this study uniquely integrates systemic data with kidney biopsies, offering a comprehensive analysis of the potential link among oxidative fibrinogen alternations, SLE renal involvement, and atherothrombotic risk. We confirmed increased p22phox expression (an essential subunit of NADPH oxidase complex) in kidney biopsies from patients with SLE with active lupus nephritis, particularly within CD15^+^ neutrophils infiltrates. These findings expand current understanding on the multifaced role of oxidative stress in SLE, suggesting that neutrophils might represent a key link between fibrinogen modification, lupus nephritis, and atherothrombotic events in SLE.

The association of neutrophil‐derived products with lupus nephritis is already described in literature.^47^ Namely, decreased NET degradation has been associated with glomerulonephritis, as well as with low complement levels, more severe disease manifestations, and elevated autoantibodies titers in SLE.^48^ Also, serum levels of NET remnants (elastase‐DNA and HMGB1‐DNA complexes) have been associated with worse renal outcomes in patients with active lupus nephritis.^49^ Our findings suggest that these neutrophil‐derived components may not only drive local tissue damage but also induce systemic prothrombotic changes via fibrinogen alterations. Overall, we could speculate that, in SLE, neutrophils infiltration in the kidney promotes lupus nephritis through the release of ROS and NETs; concomitantly, neutrophil‐derived ROS alter fibrinogen structure and function, contributing to the heightened thrombotic risk.

This study has limitations. First, we did not directly investigate the mechanistic link between fibrinogen alterations and incidence of atherothrombotic events in SLE. Future preclinical studies are needed to further support these findings, providing mechanistic insights through animal models. Additionally, disease activity was assessed at a single timepoint (ie, at time of blood sample collection). Although ROS levels can quickly rise with high disease activity, fibrinogen oxidative modifications may take longer to occur (or to reverse), which makes it more challenging to assess their correlation with point‐in‐time disease activity scores. Furthermore, a key limitation of our renal analysis is the lack of kidney biopsy samples from healthy individuals, which precluded direct comparison of p22phox expression between SLE‐affected and nondiseased renal tissue. Nevertheless, our study offers the first demonstration that ROS‐induced fibrinogen modifications profoundly alter clot structure and function, pointing to oxidative stress as a key pathogenetic mechanism in SLE‐associated thrombosis and a potential therapeutic target for future interventions.

## AUTHOR CONTRIBUTIONS

All authors contributed to at least one of the following manuscript preparation roles: conceptualization AND/OR methodology, software, investigation, formal analysis, data curation, visualization, and validation AND drafting or reviewing/editing the final draft. As corresponding author, Dr Becatti confirms that all authors have provided the final approval of the version to be published and takes responsibility for the affirmations regarding article submission (eg, not under consideration by another journal), the integrity of the data presented, and the statements regarding compliance with institutional review board/Declaration of Helsinki requirements.

## Supporting information


**Disclosure Form**:

**Data S1** Supporting Information
