# Supplementary material for: Reactive Oxygen Species–Induced Modifications of Fibrin Clots as a Link Between Immune Responses and Atherothrombosis in Systemic Lupus Erythematosus
Source: Arthritis Rheumatol. 2025 Dec 28;78(2):344–56. doi: 10.1002/art.43371 (PMC12936896; doi:10.1002/art.43371)
Supplement: Supplementary file 2 — Data S1 Supporting Information [file ART-78-344-s002.docx]

**SUPPLEMENTAL DATA**

***Table S1.*** *Primary antibodies used for the analysis of histological sections from biopsy samples.*

| **Antibody** | **Dilution** |
| --- | --- |
| **For lymphocyte subsets** |  |
| anti-CD4 (Novocastra; Buccinasco, Italy, NCL-L-CD4-368) | 1:20 |
| anti-CD8 (Abcam, Cambridge, UK; ab17147), | 1:50 |
| anti-CD20 (Abcam, Cambridge, UK, ab9475) | 1:50 |
| anti-CD138 (Abcam, Cambridge, UK, ab34164) | 1:100 |
| **For mature neutrophils** |  |
| anti-CD15 (Abcam, Cambridge, UK, ab188610) | 1:50 |
| **For histiocytes** |  |
| anti-CD68-KP1(Abcam, Cambridge, UK, ab955) | 1:200 |
| **For myofibroblasts** |  |
| anti-alpha SMA (Abcam, Cambridge, UK, ab7817) | 1:150 |

The reactions were revealed using a tetramethylrhodamine probe (tetramethylrhodamine goat anti-mouse IgG, Invitrogen, Carlsbad, CA, code T2762), and by a fluorescent probe (goat anti-mouse IgG, FITC Conjugated, Chemicon, Billerica, MA, code AP124F).

***Table S2.*** *Comparison of blood redox parameters among patients with inactive, low disease activity and moderate-to-high disease activity.*

|  | **Median levels of the assessed parameters (IQR) in SLE patients stratified according to disease activity** | | |  | **Correlation between the parameter and SLEDAI score** |
| --- | --- | --- | --- | --- | --- |
|  | **SLEDAI**  **0**  **n=38 (%)** | **SLEDAI**  **1-5**  **n=77 (%)** | **SLEDAI**  **6+**  **n=29 (%)** | **p-value^#^** | **Kendall’s τb*^§^*** |
| Fibrinogen degradation, | 66.5 (41-89) | 66 (42-79) | 65 (50-78) | 0.878 | -0.020 (p=0.751) |
| Lymphocyte ROS | 823 (672-1087) | 832 (676-1076) | 1000 (871-1205) | 0.050* | 0.113 (0=0.063) |
| Neutrophil ROS | 2175 (1732-2833) | 2200 (1961-2832) | 2502 (2154-2996) | 0.131 | 0.127 (p=0.036*) |
| Monocyte ROS | 1592.5 (1319-2186) | 1669 (1374-2104) | 1863 (1699-2280) | 0.099 | 0.147 (p=0.016*) |
| Plasma lipid peroxidation | 1.16 (0.88-1.66) | 1.46 (1.12-1.70) | 1.36 (1.18-1.75) | 0.145 | 0.044 (p=0.470) |
| Plasma total antioxidant capacity | 16.4 (14.3-18.7) | 15.0 (12.9-17.5) | 16.3 (13.5-20.3) | 0.145 | -0.043 (0.481) |
| Fibrinogen intrinsic fluorescence | 117.5 (74-232) | 160 (100-276) | 165 (98-211) | 0.346 | 0.046 (p=0.451) |
| Fibrinogen oxidation (dityrosine content) | 296 (259-318) | 290 (250-331) | 295 (267-336) | 0.803 | 0.030 (p=0.625) |
| Lag Phase | 8.2 (5.5-12.5) | 6.5 (4.5-12.3) | 8.5 (6.5-11.7) | 0.415 | -0.0045 (p=0.942) |
| V max | 0.002 (0.001-0.003) | 0.002 (0.001-0.003) | 0.002 (0.001-0.002) | 0.634 | 0.045 (p=0.457) |
| Max Abs | 0.12 (0.08-0.15) | 0.11 (0.09-0.14) | 0.12 (0.11-0.13) | 0.763 | 0.042 (p= 0.487) |

*§ from Kendall’s coefficient of rank correlation*

*# from Kruskal-Wallis test comparing the three groups of SLE patients with no, low and moderate-to-high disease activity.*

**statistically significant for p<0.05*

*IQR: interquartile range; ORAC: oxygen radical absorbance capacity; ROS: reactive oxygen species; SLEDAI: systemic lupus erythematosus disease activity index; TBARS: thiobarbituric acid reactive substance.*

***Table S3.*** *Comparison of blood redox parameters among patients with vs without history of cardiovascular events*

|  | **In patients with history of cardiovascular events (n=33)** | **In patients without history of cardiovascular events (n=111)** | **p-value^#^** |
| --- | --- | --- | --- |
|  | **Median levels (IQR)** | |  |
| Fibrinogen degradation | 68 (54-79) | 65 (41-81) | 0.417 |
| Lymphocyte ROS | 900 (798-1147) | 834 (668-1122) | 0.131 |
| Neutrophil ROS | 2502 (2087-2924) | 2230 (1833-2905) | 0.119 |
| Monocyte ROS | 1819 (1573-2256) | 1684 (1346-2186) | 0.236 |
| Plasma lipid peroxidation | 1.55 (1.16-1.95) | 1.33 (1.02-1.69) | 0.112 |
| Plasma total antioxidant capacity | 16.8 (13.6-20.3) | 15.7 (13.2-18.0) | 0.175 |
| Fibrinogen intrinsic fluorescence | 175 (106-276) | 137 (89-241) | 0.327 |
| Fibrinogen oxidation (dityrosine content) | 283 (253-331) | 297 (256-327) | 0.333 |
| Lag Phase | 9.5 (5.5 – 12.3) | 7.7 (4.5 – 12.5) | 0.253 |
| V max | 0.002 (0.001-0.003) | 0.002 (0.001-0.003) | 0.644 |
| Max Abs | 0.12 (0.10-0.15) | 0.12 (0.08-0.14) | 0.430 |

*# from Mann-Whitney test comparing the two groups of SLE patients with vs without history of cardiovascular events.*

*Table* ***S4****. Colocalization of the p22phox NADPH oxidase subunit with different cell types*

| Cell type | Fraction of red overlapping green  M_1_ | Fraction of green overlapping red  M_2_ |
| --- | --- | --- |
| CD15 | 0.42 ± 0.10 | 0.76 ± 0.13 |
| CD138 | 0.12 ± 0.01 | 0.43 ± 0.09 |
| CD8 | 0.36 ± 0.21 | 0.53 ± 0.27 |
| CD20 | 0.06 ± 0.06 | 0.21 ± 0.04 |
| CD68 | 0.20 ± 0.01 | 0.43 ± 0.09 |
| CD4 | 0.37 ± 0.08 | 0.36 ± 0.06 |
| Alpha-SMA | 0.20 ± 0.03 | 0.32 ± 0.07 |

Quantitative colocalization analyses were performed using the ImageJ JACoP plugin. The thresholded overlap coefficient is reported along with its parameters: M1 (fraction of red fluorescence overlapping green fluorescence) and M2 (fraction of green fluorescence overlapping red fluorescence).

***Supplemental figure 1***

***
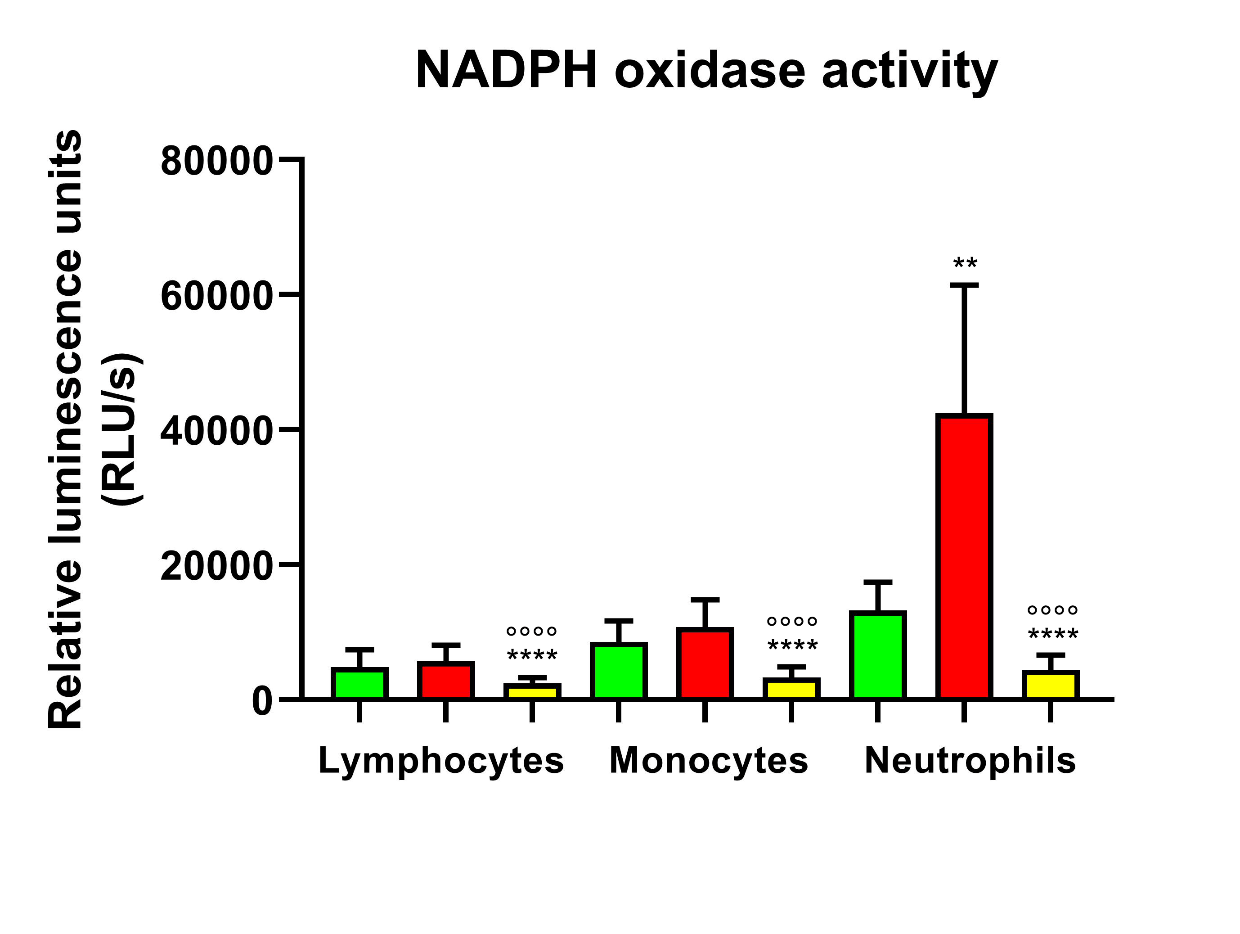
***

NADPH oxidase activity was assessed by luminometric assay in lymphocytes/monocytes/neutrophils from SLE patients and controls. All assays were performed in the absence and in the presence of a specific NADPH oxidase inhibitor (apocynin).

*Statistical significance is indicated as follows: * (vs. Control), ° (vs. SLE), with corresponding thresholds defined as p < 0.05 (*), p < 0.01 (**), p < 0.001 (***), and p < 0.0001 (****).*

***Supplemental figure 2***


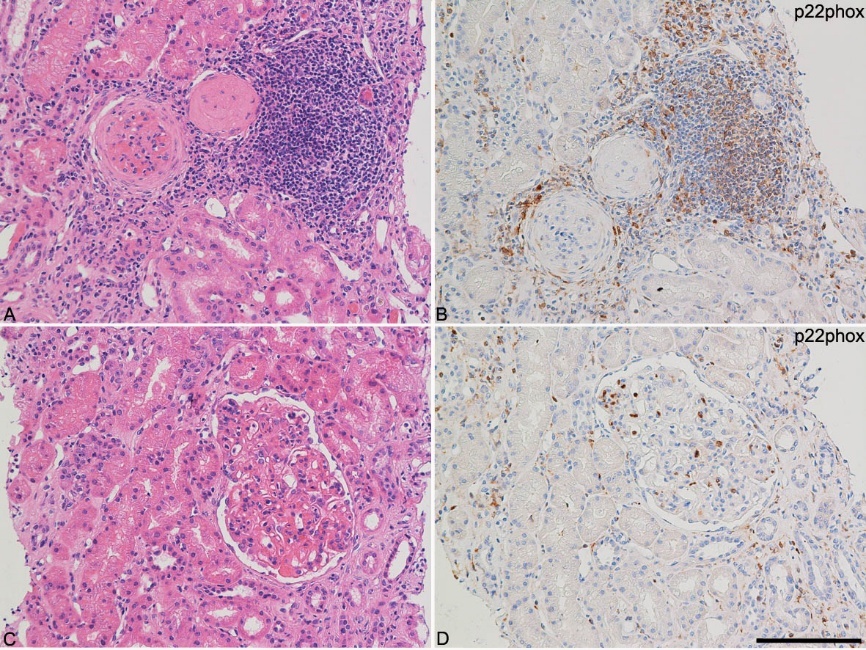


Representative renal biopsy from a patient with lupus nephritis. Representative renal biopsy from a patient with lupus nephritis and p22phox detection (**A)**. Medium-power view of a renal biopsy specimen from a patient with lupus nephritis, showing sclerotic glomeruli surrounded by an inflammatory infiltrate (**B)**. On immunohistochemistry, some inflammatory cells are immunoreactive for p22phox (**C)**. Medium-power view of a renal biopsy specimen from a patient with lupus nephritis showing a glomerulus with minor changes and sparse inflammatory cells, some of which are immunohistochemically positive for p22phox (**D**). Staining. A and C: hematoxylin-eosin. B and D immunohistochemistry was revealed by 3,3’-diaminobenzidine (DAB) and mildly counterstained by Harris hematoxylin. Scale bar: 150 µm

***Supplemental figure 3***


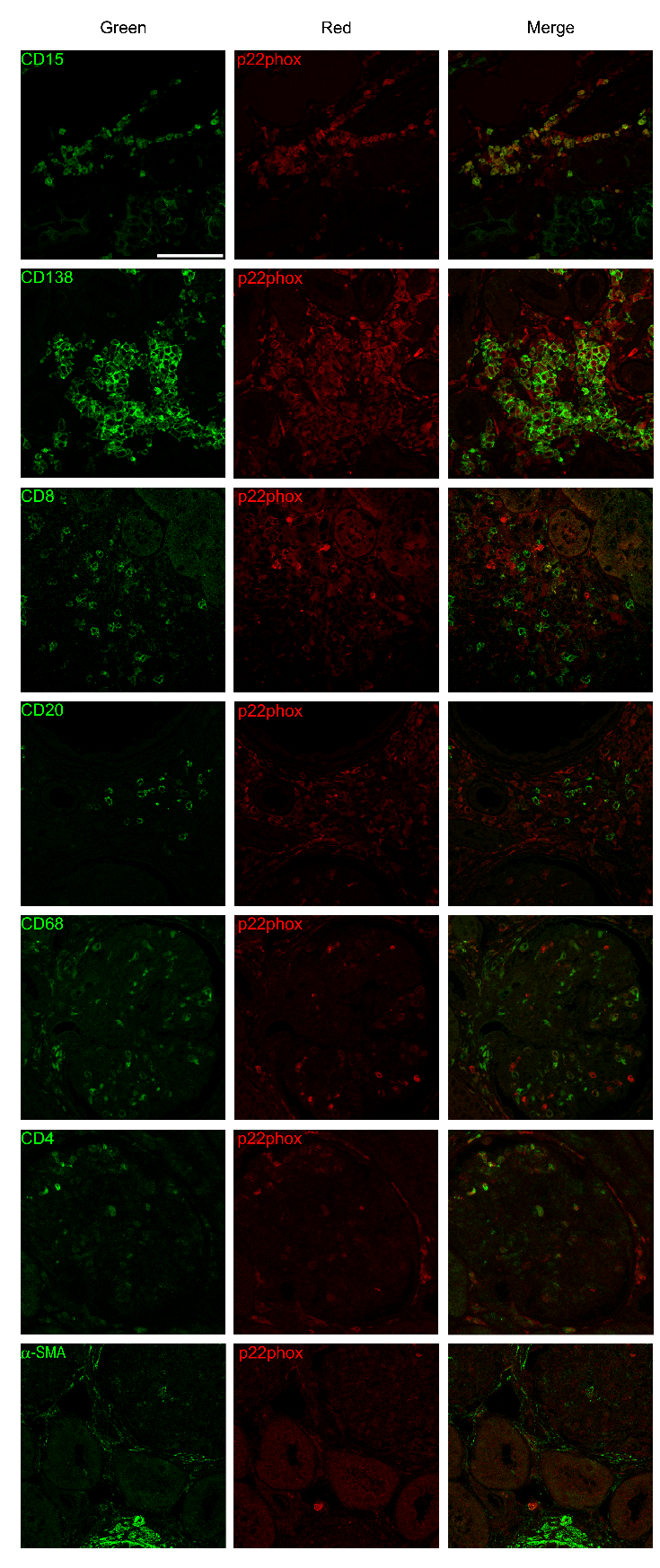


Confocal microscopy analysis showing p22phox immunoreactivity in various renal cell populations, including CD15⁺ neutrophilic granulocytes, CD138⁺ plasma cells, CD8⁺ cytotoxic T lymphocytes, CD20⁺ B lymphocytes, CD68⁺ monocytes/macrophages, CD4⁺ T helper lymphocytes, and α-SMA⁺ mesangial or myofibroblast-like cells. Scale bar: 40 µm

***Supplemental figure 4***

**
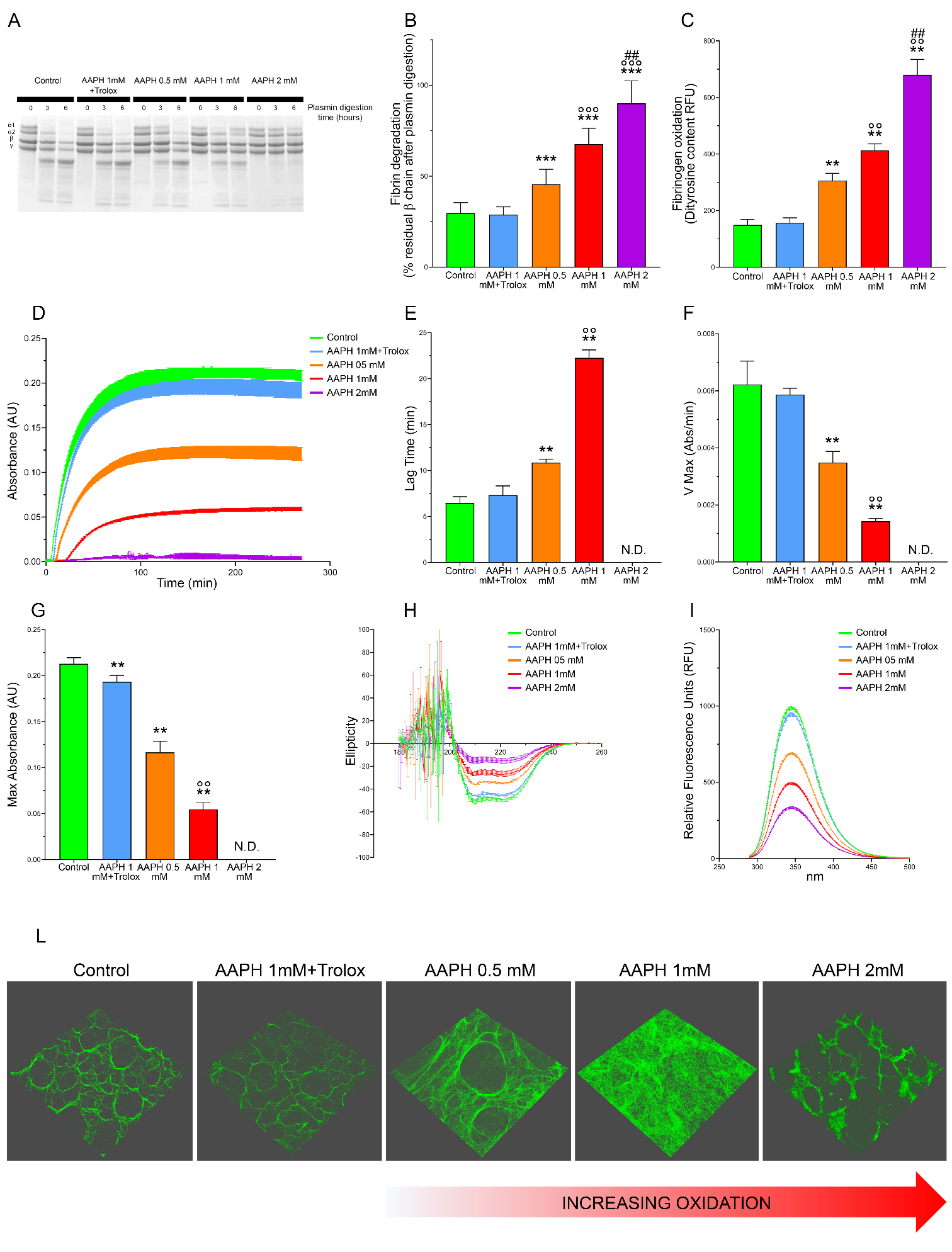
**

Effects of oxidative stress on fibrinogen structure and function. To evaluate the effect of fibrinogen oxidation on fibrin formation, fibrin degradation and fibrinogen structure alterations, human fibrinogen (Sigma, Milan, Italy) was incubated with increasing concentrations of the peroxyl radical generator 2,2′-azobis(2-amidinopropane) dihydrochloride (AAPH), in the absence or presence of the antioxidant Trolox. **(A)** Representative gel of fibrin degradation after 0, 3, and 6 hours of plasmin digestion. Values are reported as mean±SD in **B**. **(C)** Fibrinogen oxidation was evaluated by dityrosine formation. **(D)** Representative curves of thrombin-catalyzed fibrinogen polymerization and corresponding Lag Time **(E)**, Vmax **(F)**, and Max absorbance **(G)**. **(H)** Fibrinogen secondary structure was evaluated by far-UV circular dichroism. Intrinsic emission fluorescence spectroscopy **(I)** was used to investigate changes in protein tertiary structure and to estimate their microenvironment and the amount of present tryptophan residues. **(L)** Three-dimensional confocal microscopy images of fibrin gels (630× magnification).

*Statistical significance is indicated as follows: * (vs. Control), ° (vs. AAPH 0.05 mM), and # (vs. AAPH 1 mM), with corresponding thresholds defined as p < 0.05 (*), p < 0.01 (**), p < 0.001 (***), and p < 0.0001 (****).*

**Supplemetary Methods**

**Renal biopsy analysis and p22phox detection**

Ten kidney biopsies from patients with lupus nephritis were retrospectively collected from the hospital pathology files. The available histopathological slides were reviewed by an expert pathologist to confirm the original assessment. We obtained further unstained 3-4 μm-thick histological sections from the paraffin tissue blocks to perform immunohistochemistry and immunofluorescence analysis by confocal microscopy. Immunofluorescence and immunohistochemical methods have been previously described^1^.

To explore the tissue expression of NADPH oxidase, we tested the tissue expression of p22phox, a critical component of the superoxide-generating NADPH oxidase system^2^. An anti-cytochrome b245 Light Chain/p22-phox (Abcam, Cambridge, UK, ab75941; dilution 1:50) primary antibody was immunohistochemically tested to confirm the expression of this enzyme. Then, confocal microscopy analysis was carried out to explore which cells expressed p22phox. Each histological section treated with the primary antibody was also incubated with the antibodies reported in **Table S1**. Immunoreactivity was analyzed by confocal microscopy (LSM 510 Meta scan head integrated with the Axiovert 200 M inverted microscope; Carl Zeiss, Jena, Germany) with a ×63 oil objective. Images were acquired in multi-track mode, using consecutive and independent optical pathways.

Quantitative colocalization analyses were performed using the JACoP Plugin available in Fiji ImageJ, developed by the Bioimaging and Optics Platform (BIOP) for colocalization analysis of confocal microscopy images^3,4^. The Manders coefficient is a widely used metric in colocalization studies, providing a quantitative measure of the overlap between two fluorescence signals in microscopy datasets^5^. In our analysis, thresholded Manders’ coefficients M1 and M2 were calculated to evaluate signal co-occurrence. Specifically, M1 represents the fraction of the red signal (p22phox) overlapping with the green signal (cells), while M2 indicates the fraction of the green signal (cells) overlapping with the red (p22phox). These coefficients quantify the extent to which the two fluorescent signals are spatially coincident.

1. Nicastro M, Vescovini R, Maritati F, et al. Fibrocytes in Chronic Periaortitis: A Novel Mechanism Linking Inflammation and Fibrosis. Arthritis Rheumatol 2019;71(11):1913-22.

2. Ushio-Fukai M, Zafari AM, Fukui T, Ishizaka N, Griendling KK. p22phox is a critical component of the superoxide-generating NADH/NADPH oxidase system and regulates angiotensin II-induced hypertrophy in vascular smooth muscle cells. J Biol Chem 1996;271(38):23317-21.

3. Liang W, Sagar S, Ravindran R, et al. Mitochondria are secreted in extracellular vesicles when lysosomal function is impaired. Nat Commun 2023;14(1):5031.

4. Schindelin J, Arganda-Carreras I, Frise E, et al. Fiji: an open-source platform for biological-image analysis. Nat Methods 2012;9(7):676-82.

5. Aditya V, Tambe V, Yue W. Development of a Novel Automated Workflow in Fiji ImageJ for Batch Analysis of Confocal Imaging Data to Quantify Protein Colocalization Using Manders Coefficient. Bio Protoc 2025;15(7):e5285.
